# Vascular depression consensus report – a critical update

**DOI:** 10.1186/s12916-016-0720-5

**Published:** 2016-11-03

**Authors:** Howard J. Aizenstein, Andrius Baskys, Maura Boldrini, Meryl A. Butters, Breno S. Diniz, Manoj Kumar Jaiswal, Kurt A. Jellinger, Lev S. Kruglov, Ivan A. Meshandin, Milija D. Mijajlovic, Guenter Niklewski, Sarah Pospos, Keerthy Raju, Kneginja Richter, David C. Steffens, Warren D. Taylor, Oren Tene

**Affiliations:** 1 Department of Psychiatry, University of Pittsburgh Medical Center, Pittsburgh, PA USA; 2 Memory Disorders Clinic, Riverside Psychiatric Medical Group, Riverside, CA USA; 3 Department of Psychiatry, Columbia University, New York, NY USA; 4 Division of Molecular Imaging and Neuropathology, New York State Psychiatric Institute, New York, NY USA; 5 Department of Psychiatry, University of Pittsburgh Medical School, Pittsburgh, PA USA; 6 Department of Psychiatry and Behavioral Sciences, University of Texas Health Science Center at Houston, Houston, TX USA; 7 Division of Molecular Imaging and Neuropathology, New York State Psychiatric Institute, Columbia University, New York, NY USA; 8 Institute of Clinical Neurobiology, Alberichgasse 5/13, Vienna, A-1150 Austria; 9 Department of Geriatric Psychiatry of the St. Petersburg Psychoneurological Research Institute named after V. M. Bekhterev, Medical Faculty of St. Petersburg University, St. Petersburg, Russia; 10 Clinical Department, Scientific and Practical Center of Psychoneurology named after V. M. Soloviev, St. Petersburg, Russia; 11 Neurology Clinic, Clinical Center of Serbia, School of Medicine University of Belgrade, Belgrade, Serbia; 12 University Clinic for Psychiatry and Psychotherapy, Paracelsus Private Medical University, Nuremberg, Germany; 13 Consultant in Old Age Psychiatry, Cheshire and Wirral Partnership NHS Foundation Trust, Chester, UK; 14 Faculty for Social Sciences, Technical University of Nuremberg Georg Simon Ohm, Nuremberg, Germany; 15 Department of Psychiatry, University of Connecticut Health Center, Farmington, CT USA; 16 Department of Psychiatry, The Center for Cognitive Medicine, Vanderbilt University Medical Center, Nashville, TN USA; 17 Department of Veterans Affairs Medical Center, The Geriatric Research, Education, and Clinical Center (GRECC), Tennessee Valley Healthcare System, Nashville, TN USA; 18 Departments of Neurology and Psychiatry, Tel Aviv Medical Center, Tel Aviv, Israel; 19 Tel Aviv University, Sackler Faculty of Medicine, Tel Aviv, Israel

**Keywords:** Late-life depression, Vascular depression, Structural neuroimaging, Cerebrovascular lesions, White matter lesions, Clinicopathological correlations, Peripheral markers, Neuropathology

## Abstract

**Background:**

Vascular depression is regarded as a subtype of late-life depression characterized by a distinct clinical presentation and an association with cerebrovascular damage. Although the term is commonly used in research settings, widely accepted diagnostic criteria are lacking and vascular depression is absent from formal psychiatric manuals such as the Diagnostic and Statistical Manual of Mental Disorders, 5^th^ edition – a fact that limits its use in clinical settings. Magnetic resonance imaging (MRI) techniques, showing a variety of cerebrovascular lesions, including extensive white matter hyperintensities, subcortical microvascular lesions, lacunes, and microinfarcts, in patients with late life depression, led to the introduction of the term “MRI-defined vascular depression”.

**Discussion:**

This diagnosis, based on clinical and MRI findings, suggests that vascular lesions lead to depression by disruption of frontal–subcortical–limbic networks involved in mood regulation. However, despite multiple MRI approaches to shed light on the spatiotemporal structural changes associated with late life depression, the causal relationship between brain changes, related lesions, and late life depression remains controversial. While postmortem studies of elderly persons who died from suicide revealed lacunes, small vessel, and Alzheimer-related pathologies, recent autopsy data challenged the role of these lesions in the pathogenesis of vascular depression. Current data propose that the vascular depression connotation should be reserved for depressed older patients with vascular pathology and evident cerebral involvement. Based on current knowledge, the correlations between intra vitam neuroimaging findings and their postmortem validity as well as the role of peripheral markers of vascular disease in late life depression are discussed.

**Conclusion:**

The multifold pathogenesis of vascular depression as a possible subtype of late life depression needs further elucidation. There is a need for correlative clinical, intra vitam structural and functional MRI as well as postmortem MRI and neuropathological studies in order to confirm the relationship between clinical symptomatology and changes in specific brain regions related to depression. To elucidate the causal relationship between regional vascular brain changes and vascular depression, animal models could be helpful. Current treatment options include a combination of vasoactive drugs and antidepressants, but the outcomes are still unsatisfying.

## Background

Depressive symptoms in the elderly are common; subsyndromal depression rates in community-dwelling older adults are estimated at 12–30 %, compared with 2–5 % for major depressive disorder (MDD) as defined in the Diagnostic and Statistical Manual of Mental Disorders, fourth edition, text revised (DSM-IV-TR) [1–3]. Although the risk of a depressive episode in the elderly is usually lower than that observed in younger adults [4], the consequences and prognosis of depression in an older population are usually worse. Increasing age in depressed persons accounts for an unfavorable clinical course with higher relapse rates [5], worse treatment response, and incomplete functional recovery [6].

Depression in the elderly is often referred to as late-life depression (LLD), commonly defined as any depressive episode occurring at age 65 or later, regardless of age of onset. LLD can either be late-onset depression (LOD), when the first lifetime depressive episode began after age 65 (some studies place this cut-off at 50 or 60 years of age). In contrast, early-onset depression (EOD) means that an older adult has experienced recurrent depressive episodes with a first episode occurring earlier in life. LLD is of great interest because of its clinical significance and complex basis, which may affect the outcome in the depressed elderly and increase the risk of cognitive impairment and poor quality of life [7–10].

**Fig. 1:**
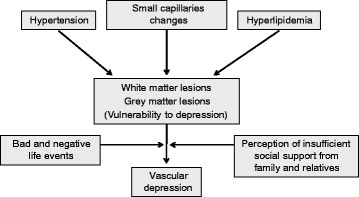
Flow chart of possible mechanism of vascular depression (adapted from [233])

In contrast to depressive disorders in younger adults, LLD is associated with cerebrovascular comorbidities and microvascular lesions, as represented in particular by white matter hyperintensities (WMHs) on structural magnetic resonance imaging (MRI), subcortical lacunes, microinfarcts and microbleeds, but also frontal and temporal (hippocampal) gray matter changes/atrophy, neurodegenerative pathologies, and related biochemical changes [11]. Due to its “organic basis”, these etiological factors were used to identify the disorder – through the old concept of “atherosclerotic depression” [12] to the more recent term of “vascular depression” (VaDep). In 1997, Alexopoulos et al. [13] suggested the “VaDep hypothesis”, which argues that cerebrovascular disease (CVD), including small vessel ischemic changes, may predispose, precipitate, or perpetuate some geriatric depressive symptoms as a consequence of structural damage to frontal–subcortical circuits, with disruption of cortico–striato–pallido–thalamo–cortical pathways as their underlying systems [13–16] (Fig. 1). Newer MRI based studies argue that VaDep accounts for up to 50 % of MDD cases in the elderly [17]. Boosted by modern neuroimaging techniques, Krishnan et al. [18] coined the entity of “MRI-defined VaDep”, which by definition includes CVD findings on MRI. Patients with VaDep were suggested to have a distinct clinical and neuropsychological profile and a positive association with hypertension [19], supporting the notion that VaDep represents a unique and valid subtype of LLD [20–22], although this has not been confirmed by others [23–27]. The MRI literature supporting the VaDep hypothesis shows that loss of brain volume and white matter integrity are associated with poor clinical treatment outcomes [28, 29]. Individuals with VaDep are at greater risk to develop cognitive impairment, more likely related to vascular dementia than to Alzheimer’s disease (AD) [30]. However, recent data showed that VaDep is not a risk factor for AD [31, 32], although older cognitively unimpaired patients with depressive episodes may have more underlying AD pathology, in particular β-amyloid deposition [33, 34]. In general, depression in vascular dementia is clinically different from that in AD [30]. Although MDD is mainly diagnosed, treated, and studied by psychiatrists, the DSM-IV-TR, and the newer DSM-V, do not acknowledge the diagnosis of VaDep and do not address its treatment-resistant course. In addition, MRI is usually used to rule out organic causes for psychiatric symptoms rather than to validate a psychiatric diagnosis. Thus, it is clear why the definition of this entity and agreed diagnostic criteria remain elusive, a fact that complicates studies in this field and the introduction of therapeutic options.

The relationship between structural and biochemical cerebral changes contributing to brain network dysfunctions in VaDep is also not fully understood, and recent neuropathological findings even challenged the validity of the VaDep hypothesis (for a review, see [31]).

The aims of this Consensus Report are to examine the current evidence for the neurobiology of VaDep. It focuses on neuroimaging and neuropathological data to consider the relevance of cerebrovascular changes in the pathogenesis of LLD, but also considers the role of peripheral markers in VaDep, the differences in biological substrates based on age of depression onset, and the therapeutic options. Based on this overview, we present ideas about future research in this still incompletely elucidated area of VaDep and make proposals for future studies in order to clarify the relations between LLD and CVD that may promote further consensus and approval of VaDep; some of these issues have been reviewed recently [7, 9, 17, 28, 31, 35–42]. The issue of post-stroke depression will not be discussed, since it has been reviewed by a different consensus group.

## Methods

Using a comprehensive search of PubMed (MEDLINE) from January 1990 until November 30, 2015, the current literature was critically reviewed on the association between VaDep and microvascular burden, gray and white matter lesions, and other structural brain changes resulting in brain network dysfunction detected by MRI, as well as neuropathological studies.

Approximately 4000 articles were reviewed, but studies were only included if they satisfied the following criteria: (1) the patient population had a diagnosis of LLD and/or VaDep, (2) diffusion tensor imaging was the imaging technique used, (3) contained a vascular depression hypothesis, and (4) publication in English. Additionally, references from the selected papers were evaluated and included if they were found to be relevant to the focus of this systematic review. Exclusion criteria were publication prior to 1990, and articles discussing post-stroke depression, depression in AD, vascular dementia, and other dementias. Furthermore, the findings by the members of the consensus report summarized in the abstracts of the 9th International Congress on Vascular Dementia 2015 were included in order to supplement the data in the literature. To ensure the quality, a face-to-face meeting of the group of multidisciplinary experts (BSD, MKJ, KAJ, MDM, KR, TO) was followed by extensive e-mail correspondence among the larger group of co-authors. In order to actualize this report, the relevant literature until August 30, 2016, was included.

## Results

### Clinical features of VaDep

There is considerable evidence to suggest that the clinical manifestations of VaDep are distinct from non-VaDep in the elderly (Table 1). This may be related to differences in age of depression onset [20, 43, 44], as individuals with VaDep tend to have a later age of initial onset of depression [19]. However, even individuals with EOD may be at risk of transitioning to VaDep since some studies have implied a bi-directional link between vascular disease and depression [45, 46].

**Table 1:** Clinical features of vascular depression (VaDep) and non-VaDep

Clinical features of VaDep	Clinical features of non-VaDep
Depression occurring at age 65 years or later	Depression occurring at age 50 to 60 years
Absence of family history	Occasional family history
Executive dysfunctions, loss of energy, subjective feeling of sadness, anhedonia, psychomotor retardation, motivational problems, reduced processing speed and visuospatial skills, deficits in self-initiation, lack of insight; depressive symptomatology may not meet criteria for any mood disorder requested in DSM-V	Sadness, depression according to DSM-V diagnostic criteria, increased suicidality, reduced verbal fluency
Higher cardiac illness burden, increased rates of vascular risk factors (hypertension, etc.)	Lower or same cardiac illness burden and rates of vascular risk factors (hypertension, etc.)
Higher risk for cognitive decline and progression to dementia	Lower or similar risk for cognitive decline and progression to dementia
Fluctuating course of cognitive impairment due to progression of white matter hyperintensities	
Greater treatment resistance and poorer outcome	Lower or same treatment resistance and outcome(?)
Associated with increased mortality	

The clinical presentation of VaDep is characterized by psychomotor slowing, lack of initiative and apathy, absence of a family history of depression, and a medical history of hypertension. Cognitive impairment is also common, particularly executive dysfunction and impaired processing speed. Functional disability may also be disproportional to the severity of cognitive impairment. Overall, patients with VaDep have greater cognitive impairment and disability than individuals of the same age with non-vascular depression [16, 19, 28, 36, 37, 47, 48].

Observations of VaDep being associated with greater disability, poorer outcomes, and executive dysfunction [20, 21, 49] led to a reconceptualization of VaDep, with subsequent proposals of the “depressive–executive dysfunction syndrome” [37, 50, 51] or “depression–cognitive impairment disease” [52]. However, although these conceptualizations overlap, they are not necessarily synonymous. In depressed older patients, cerebral vascular burden is related to slower processing speed even in the absence of manifest generalized vascular disease [53]. Depressed older people with lacunar infarcts in deep white matter are characterized by more “motivational” problems than those with no vascular disease [41, 54]. A comparison of subcortical ischemic depression and depressive–executive dysfunction syndrome showed no significant differences in predicting functional disability [55].

Other clinical factors distinguish VaDep from non-vascular depression, including age, higher cardiac illness burden, and greater deficits in depression symptoms of self-initiation and concentration [56], while loss of libido, agitation, risk of suicidal activity, and a family history of mental illness, were associated with EOD and not specifically with VaDep [19]. While loss of energy and lethargy, apathy, and executive dysfunction were frequently observed in patients with VaDep [57], other symptoms, namely psychomotor retardation and anhedonia, were not significantly associated with vascular risk factors [24]. However, suicidal patients scored higher in the vascular cumulative illness rating scale [58]. There may also be population differences. In a multiethnic clinical sample VaDep was overrepresented among African Americans, probably due to higher rates of cardiovascular disease, hypertension, and stroke [59].

This is concordant with other work associating increased risk of depression in populations with vascular disease. For example, people with peripheral artery disease have a higher incidence of depressive symptoms than those without. Further, in peripheral artery disease, depressive symptoms are associated with increased cardiovascular mortality [60]. Depression and vascular disease are both common among the elderly, and cardiac dysfunction in association with depression is well documented [46, 61, 62]. These observations may provide clues about the mechanistic relationships, as depression-associated changes of peripheral vascular resistance are essential for the association of MDD with cardiovascular disease [63, 64].

Depression may also contribute to adverse vascular health outcomes. There is a strong association between baseline depression scores and later cardiovascular mortality in hypertensive elderly people [65–68]; depressive states are considered a potential CVD mortality risk factor [69]. Meta-analyses of longitudinal cohort and case–control studies reporting depression at baseline and cardiovascular disease outcomes at follow-up identified MDD as the most important risk factor for developing cardiovascular disease, although this evidence is related to a high level of heterogeneity [70]. Depression-like behaviors have been observed in a rat model of chronic cerebral hypoperfusion [71]. Depression is a strong risk factor for stroke in middle aged women [72], and elderly patients with high levels of depressive symptoms showed an increased risk of stroke events [62]. Unsurprisingly, as accepted diagnostic criteria for VaDep are lacking, there is no epidemiological outcome data about VaDep in the general population.

While loss of energy and lethargy, apathy, and executive dysfunctions are frequently observed in patients with VaDep [57], other symptoms, namely psychomotor retardation and anhedonia, are not significantly associated with vascular risk factors [24]. However, suicidal patients scored higher in the vascular cumulative illness rating scale [58].

### VaDep and cognitive impairment

Depressive symptoms in old age flag an increased likelihood of cognitive decline in later life [10, 73]; the effect is particularly high in individuals with depression and vascular disorder [9]. Depression and vascular disorder are highly prevalent among elderly subjects with mild cognitive impairment (MCI) and in cognitively normal elders, with an increased risk of developing MCI [74], particularly in those with higher cerebral amyloid burden [75]. Recent meta-analyses showed that LLD increases the risk of AD by 65 % and vascular dementia by 150 % compared to non-depressed older adults [30]. The relationship between LLD and risk of dementia is also particularly relevant among older adults with MCI, since the co-occurrence of depressive symptoms and MCI leads to an increased risk of dementia by approximately 30 % compared to MCI individuals with no depression [76].

Nevertheless, not only the presence of depressive symptoms is important to determine the risk of dementia in LLD/VaDep. Recent longitudinal studies, with up 10 years of follow-up, demonstrated that persistently high levels or increasing levels of depressive symptoms are the most important predictors of dementia in LLD [77, 78]. The association with depressive symptoms is stronger for the MCI subtype with memory impairment [79], but this was independent of underlying vascular disease [80–82]. Some studies suggested an association between white matter microstructural damage and depressive symptoms in MCI patients with small vessel disease [74, 83], whereas others showed no association between depressive symptoms and the rate of incident MCI [84]. The path from CVD to VaDep and to vascular dementia appears to be likely reciprocal and not direct or sequential [85], whereas others suggested that depression is an independent risk factor for subsequent vascular dementia [86]. There are multiple pathways to poor cognitive outcomes and therefore the relationship reflecting either a causal effect of depression on cognitive decline, or a common cause, or both, should be further explored [25, 87]. Older patients with greater WMH volume appear to progress to dementia at a similar rate as those who were never depressed with similar WMHs [40]. However, individuals with LLD are, in general, at greater risk of developing vascular dementia as compared to AD [30], and depression in vascular dementia is quantitatively and qualitatively different from that in AD [88]. Recent clinicopathological studies showed that depressive symptoms in old age associated with cognitive decline were independent of the neuropathologic hallmarks of dementia, and none of the neuropathological markers (CVD, AD-related pathology) were related to the level of depressive symptoms or changes in symptoms over time [89–91]. Despite frequent neuroimaging evidence in favor of a possible causal link between depressive symptoms and cognitive impairment in old age, neuropathological data showed that LLD and VaDep are not a risk factor for AD pathology and that cognitive impairment in old age may be due to a variety of pathological and molecular changes [25, 31, 32]. According to a recent clinical study, the presence of depressive symptoms in amnestic MCI patients is not predictive of conversion to dementia [92]. On the other hand, older, cognitively normal patients with depressive episodes were found more likely to have an underlying AD pathology, in particular β-amyloid deposition [33, 34]. However, there are inconsistent results for plasma and/or cerebrospinal fluid levels of soluble β-amyloid 42 in LLD [93].

### Structural brain abnormalities

Structural and functional imaging studies provide information about the underlying (micro) structural changes in VaDep, including localization and anatomical size and shape of gray and white matter lesions [94].

#### White matter lesions

MRI-defined VaDep requires evidence of cerebrovascular changes on neuroimaging, including WMHs [19]. The validity of this subtype, characterized by voxel-based morphometry and diffusion tensor imaging MRI as well as executive dysfunction, was confirmed by several studies [35, 37, 95]. Microstructural brain lesions, especially WMHs, are more frequently found in patients with LLD compared to controls [96–102]. They involve, in particular, white matter tracts underlying emotional and cognitive function, i.e., left superior longitudinal fascicle, cingulum bundle, and frontal projections to the corpus callosum [103–105], disrupting frontal and frontal-to-limbic white matter tracts [106]. The importance of deep WMHs and subcortical lacunar infarcts on the risk of depressive symptoms [107–109] and a strong relationship between depression and WMH volume [110] were emphasized. A recent study showed that diffuse WMHs are one of the major factors that cause apathy and have negative effects on quality of life [111], while others could not demonstrate any significant association between WMH progression and depression at baseline [112]. A multicenter longitudinal study showed that WMHs predated the development of depressive symptoms in later life. Greater WMH severity is a critical risk factor predicting future depression risk, which supports the VaDep hypothesis [17]. The severity of WMHs may serve as a biomarker for LLD [113], although the results of a European multicenter study (LADIS) showed that baseline severity of WMHs no longer predicted depressive symptoms at 3 years or incident depression [114]. A strong association between deep WMHs and depression compared to periventricular ones was observed [107]. A recent study showed that individuals with extensive WMHs at baseline had a high risk of developing severe depressive symptoms, with the relationship strengthening in the absence of cardiovascular disease. In contrast, when depressive symptoms or antidepressant prescription was the outcome, larger brain volume and temporal lobe volume, but not WMH, were negatively associated with the development of depression [115].

Systematic reviews reported a four-fold higher prevalence of deep and periventricular WMHs in LOD/LLD subjects than in those with EOD and healthy controls [98, 116]. Together with a more frequent presence of cardiovascular risk factors (hypertension, dyslipidemia, vascular co-morbidity, diabetes mellitus) [117–120], and a history of CVD, a higher burden of WMHs was proposed to be a diagnostic criterion for VaDep or subcortical ischemic depression [19]. Deep WMHs are associated with a more fluctuating but not more severe course of depression. Lacunar infarcts do not correlate with severity or course of depressive symptoms, while periventricular WMHs are associated with poorer executive function [54], and large confluent WMHs with cognitive impairment and disability [121]. Greater longitudinal increases in WMH volume are associated with more persistent depressive symptoms [122, 123]. All WMHs, except the least severe, have been shown to have a negative effect on depression outcome and, together, both deficits in neuropsychological function and severity of WMHs predict worse outcome [119]. The WMH volume in the frontal lobe conferred a risk of comorbid depressive disorder in AD, which implies that comorbid depression in AD may be attributed to vascular causes, and does not essentially differ from VaDep without AD-related changes [124]. Others suggested that dysfunctions in left-sided functionally salient cortical regions and relative preservation of deficit awareness, provided by the right hemisphere, may explain depressive symptoms in the initial clinical stages of AD [125].

#### Gray matter changes

Structural abnormalities in LLD also involve gray matter reduction in the bilateral orbitofrontal and medial frontal cortex, subcallosal gyrus, hippocampus, parahippocampus, amygdala, insula, and anterior cingulate cortex, and cortical thinning and volume reduction in lentiform nucleus (for a review, see [31, 32, 126, 127]), indicating that these alterations within the fronto–striato–limbic network and disrupted orbitomedial prefrontal limbic network play a key role in the pathophysiology of VaDep [47, 128–132]. In very old adults with depression, loss of grey matter volume was most significant in the bilateral insula and anterior cerebral cortex, supporting a cerebrovascular pattern of LLD [133]. These changes, together with WMHs, are associated with both depression and cognitive decline and may precede the incidence of both disorders in elders by 10 years [134], suggesting an etiological pathway from ischemia to increased depressive burden [133]. The currently largest worldwide effort to identify subcortical brain alterations showed reduction of hippocampal and amygdala volumes in recurrent and/or early onset MDDs, moderated by age of onset and first episode versus recurrent episode status [135]. Later age at onset of depressive symptoms in LLD subjects is associated with smaller left anterior cingulate thickness, and more white matter and subcortical gray matter hyperintensities [128]. The greater burden of depressive symptoms was significantly related to low fractional anisotropy in MRI of white matter underlying the right ventral anterior cingulate in depressed older adults with vascular disease [136].

The relationship between vascular disease and these findings in LLD/VaDep is not entirely clear, and both constructs apparently share many neuropathophysiological characteristics and changes, although VaDep appears more related to cerebrovascular rather than to other types of brain lesions [137–139]. Both gray and white matter abnormalities in VaDep indicate that four major neurocircuits are involved, namely default mode, cognitive control, frontolimbic, and corticostriatal networks [140–143]. The default-mode network includes several brain regions that are active during rest and inhibited during goal-directed tasks [140, 142]. It consists of the medial prefrontal cortex, posterior cingulate cortex, precuneus, and medial temporal lobe, and its functions include self-prospecting, internal monitoring, memory retrieval, future planning, and the theory of mind. Depression is associated with decreased default-mode network activity during a cognitive or emotional task or increased activity during negative rumination [142]. Specifically, in LLD, default-mode activity is increased in the subgenual cingulate and thalamus region [140].

The cognitive control network consists of the dorsolateral prefrontal cortex, the dorsal anterior cingulate cortex, and the posterior parietal cortex, and is involved in attention-dependent executive tasks such as decision-making, working memory, and task switching [142]. An impaired cognitive control network has also been associated with impaired cognition in depression [142].

The frontolimbic or affective network consists of the amygdala, the subgenual anterior cingulate cortex, hypothalamus, orbitofrontal cortex, and nucleus accumbens, with the main functions of processing emotion, regulating the emotion–mood relationship, and mediating motivated behaviors. Specifically, decreased amygdala volume, decreased orbitofrontal cortex volume, and a disrupted uncinate tract, which connects amygdala and hippocampus to the frontal centers, have been demonstrated in LLD [142].

Corticostriatal networks connect frontal regions to basal ganglia and thalamus; their function primarily includes the mediation of motor and executive control and emotional behavior [141]. However, other volumetric differences observed in LLD may be influenced by vascular disease, but could also reflect premorbid vulnerabilities or occur through other (neurodegenerative) pathways. If unrelated to vascular disease, these structural differences could thus serve as vulnerability factors contributing to the risk of depression.

#### Cerebrovascular lesions

Review studies indicate a higher frequency of depression in older people with cardiovascular disease with or without a cerebrovascular component, and suggest the possibility of a bidirectional relationship between vascular disease and depression, although the association between vascular risk factors and LLD may not be consistent [21] and the causality in the individual case may be difficult to establish. In depressed older persons, vascular burden was related to slower progressing speed also in the absence of manifest vascular disease [53]. A large body of neuroimaging data supports the notion that microvascular burden and WMHs may be key determinants of depressive episodes in late life [144]. LLD patients had a higher prevalence of silent brain infarctions, subcortical lacunes, and microbleeds, especially in the left hemisphere and in basal ganglia, compared to control groups. These lesions presented as independent risk factors for LLD [145], while microbleeds in the left hemisphere were not associated with EOD [146]. WMHs and lacunar infarcts may be non-specific vascular lesions in depressive disorders, while association of cerebral microbleeds with more severe forms of depression may indicate impaired brain iron homeostasis or episodes of cerebrovascular extraversion, which may play a role in depression etiology [147]. Microbleeds were associated with LLD but not with EOD [146]; these lesions and WMHs were associated with cerebral small vessel disease (CSVD) and reduced cerebral blood flow [148], which predicted depressive disorder in healthy older adults [149]. Depressive symptoms were seen in 10.1–39.8 % of patients with CSVD [2, 150], and patients with silent cerebral infarcts and chronic heart failure had an increased prevalence of MDD compared to those without chronic heart failure [151]; further, minor cerebrovascular incidents predisposed patients to LLD/VaDep [152]. Lacunar infarcts in deep white matter were associated with greater psychomotor retardation, motivation and energy loss, depressed mood, and cognitive decline, presumably due to disruption of frontal–subcortical networks [153, 154], while others suggested that apathy, but not depression in CSVD, is related to damage in circuits associated with emotion regulation [155]. Interestingly, while microvascular lesions tended to have a deteriorating course once diagnosed, there are no data supporting an exact parallel deterioration in VaDep symptomatology. Rather, VaDep could potentially remit with treatment, while WMHs do not (despite more than 50 % of older adults with late-life MDD failing to respond to initial treatment with first-line pharmacological therapy) [156].

### Other pathogenetic features

Recent studies using a multimodal biomarker approach have indicated relationships between depression, WMHs, and abnormalities in biomarkers related to inflammatory processes, including higher TNF receptor-2 and IL-1β levels, endothelial dysfunction, astrocytic abnormalities, platelet activation, control of blood clotting processes, lipid homeostasis, and reduced neurotrophic support, indicating the relevance of vascular disease and other factors in the pathogenesis of LLD [157–165]. Non-vascular factors may also contribute to VaDep. The same genetic, epigenetic, and environmental factors that contribute to EOD continue to confer vulnerability for depression onset in later life [166]. Although immune and endocrine disorders affect vascular risk, they may also increase the risk of depression through independent mechanisms that require further study [167]. More recently, the amyloid hypothesis of LLD was discussed [33, 34].

Recent studies have focused on the role of glia in LLD [162, 168], although the mechanisms by which glia are associated with the symptoms of MDD remain unclear. Inflammatory mechanisms and the role of cytokines and other pro-inflammatory markers have been suggested [169] (for a review, see [170]). Gliosis being reduced in EOD brains and increased in LLD suggests that subtle vascular or inflammatory changes may be important in LLD, but further studies are required to explore the complex relationship between WMHs, ischemic damages, and glial pathology in those processes. There is no evidence for a loss of serotonergic neurons or of neuritic pathology in the raphe nuclei of LLD patients [31, 171], while the mesolimbic dopamine system, especially the ventral tegmental area, involved by Lewy bodies and neurofibrillary tangles, may have an important role in LLD symptoms [172].

### Neuropathological findings in VaDep

Postmortem studies in clinically well-documented cases are crucial to elucidate the role of cerebrovascular lesions in LLD since neuropathological findings are heterogeneous [11, 31, 46, 144, 173–176]. The first report on white matter pathology in LLD [177] suggesting that white matter lesions due to microvascular dependent ischemia in the dorsolateral prefrontal cortex are important for cognitive impairment in LLD, has not been confirmed [178]. No association between depression and vascular or microvascular disease has been observed [31, 46, 95, 144, 175, 179, 180]. These results challenge the “VaDep hypothesis” by indicating that the chronic burden of microvascular lesions may not be a major pathogenic factor for LLD. Further, recent clinicopathological studies did not confirm the hypothesis that subcortical microvascular lesions and cortical microinfarcts may be essential for the development of LLD [11, 31, 95, 144, 174–176, 179–183]. There was also no confirmation for the notion that diffuse WMHs may be associated with long-term depression [184, 185] nor that general and cerebral atherosclerosis may increase the risk of incident depression in older adults (Table 2). Alternatively, it was suggested that both disorders result from a common underlying biological substrate [137]. These studies also showed no definite relationship between LLD and AD pathology, including in cerebral amyloid angiopathy [186], revealing a significant gap in our understanding of the pathobiology of LLD. It should be emphasized that the published findings in VaDep are not consistent and are often complicated by comorbid conditions, and therefore there has been limited success in demonstrating any relationship with many of these pathological changes [31, 176, 187].

**Table 2:** Negative neuropathology findings in late-life depression

Findings	Reference
No association with microvascular disease	[179]
Cerebrovascular pathology (hemorrhages, infarcts, microinfarcts, lacunes) not more severe than in non-depressed aged	[31, 175, 176, 180]
No increased white matter change	[31, 176]
No increased Alzheimer’s disease pathology	[31, 176, 180, 181]
No increased cerebral amyloid angiopathy (but association between plaque and tangle pathology and life time depression preceding Alzheimer’s disease diagnosis)	[182, 183]
No hippocampal sclerosis	[31]

### Animal models of VaDep

The chronic mild stress model of depression is well documented [188–191]; it is associated with vascular and endothelial dysfunction [192–194], both of which are risk factors for the development of cardiac disease. Further, depression-like behaviors in a rat model of chronic cerebral hypoperfusion and cerebral ischemia-induced sensitivity to depression, as well as hippocampal vascular endothelial growth factor down regulation after forced swim stress in mice, all support the clinical hypothesis of VaDep [71, 195, 196].

## Discussion

### Clinicopathological relations in VaDep

VaDep can be regarded as a distinct subtype of LLD characterized by a specific clinical presentation and an association with vascular risk factors and a variety of cerebrovascular lesions, as shown by structural MRI. The hallmark of MRI-defined VaDep is the presence of WMHs identified in T2-weighted or fluid attenuated inversion recovery sequences. These lesions, associated with CSVD, induce disruptions of frontal–subcortical pathways involved in mood regulation. WMHs are associated with advanced age, cerebrovascular risk factors (diabetes, hypertension, cardiac disease, blood pressure variability, and reduced cerebral blood flow) (for a review, see [38, 121, 197, 198]). The correlation between WMHs and altered default-mode network connectivity supported the role of vascular changes in the etiopathogenesis of VaDep [140, 145] and diminished neuropsychological performance was related to microstructural white matter abnormalities [199]. VaDep is associated with poorer endothelial function, potentially contributing to greater WMH load and basal ganglia microangiopathy [138]. WMHs in patients with LLD, especially within cortico-subcortical neural circuits, should be interpreted as the consequence of underlying microstructural dysfunctions affecting brain connectivity, mediating the association between CSVD and depression [38, 139, 200, 201], although not all studies supported the existence of WMHs as assessed by diffusion tensor imaging MRI in VaDep [202–204]. Others, however, suggested that apathy, but not depression, in CSVD is related to damage to cortical–subcortical networks associated with regulation of emotions [155].

Other frequent findings include widespread gray matter reductions related to disorders of the fronto–striato–limbic network. White matter abnormalities, particularly in the fronto–subcortical and limbic networks have been suggested to play a role in LLD even in the absence of essential gray matter changes [47, 94]. However, a recent study of MRI-defined VaDep showed that subjects with high scores of either deep WMHs or subcortical gray matter ratings had an eight-fold higher risk of developing depressive disorders in a 3-year follow-up study [17]. No association between LLD and Framingham vascular risk factors (hypertension, dyslipidemia, diabetes, etc.) was found, although positive relations between depression in elders and cardiovascular disease were observed [205, 206]. There are various mechanisms by which vascular disease may influence the development and course of depression - mechanistic disconnection, inflammation and hypoperfusion - that link underlying cerebrovascular processes with brain function influencing the development of depression [37].

### Prevention and treatment options

Brain reserve, characterized by educational attainment, may counterbalance the effect of cerebrovascular burden with respect to depressive symptoms, thereby preserving mood in late life [207]. Additionally, since older patients with both depression and vascular risk factors may be at an increased risk for functional decline, they may benefit from management of both these factors and depression [208, 209]. Overall, individuals with VaDep and deficits across cognitive domains may be at higher risk of responding poorly to selective serotonin reuptake inhibitors [119]. Positron emission tomography (PET) studies have demonstrated an increase in cortical glucose metabolism in non-demented and largely never-medicated geriatric depressed patients relative to age-matched controls in anterior and posterior cortical regions in which cerebral atrophy was observed. These regions were hypermetabolic and atrophic and were correlated with depression, which may represent a compensatory response; these findings are in contrast with the decreased metabolism observed in normal aging and neurodegenerative diseases [210]. Possible efficacy in treatment of VaDep with a combination of vasoactive and neurometabolic drugs along with several groups of modern antidepressants (selective serotonin and noradrenaline reuptake inhibitors) has been demonstrated [211]. However, no significant differences were observed in any of the neuroimaging markers (WMH accumulation) nor in treatment outcome over an interval of 12 weeks, which corresponds to the typical length of an antidepressant trial [212]. Further, no differences were found in neuropsychological factor scores [213] nor in treatment outcome between EOD and LOD subjects [128]. Neuroimaging markers may inform treatment by identifying depressed adults likely to remit with pharmacotherapy, an individualized therapeutic dose, and treatment response [29, 39]. In essence, treatment results in VaDep patients are still unsatisfying and, regardless of causal mechanisms, persons with depressive disorders and vascular disease represent a high-risk group for poor treatment response [214]. The prevalence of treatment-resistant LLD was estimated between 26 and 41 per 100 person years [215]. Greater baseline cerebrovascular risk was associated with less improvement in depression severity over time, and after controlling for co-variates, neither executive function nor processing speed predicted outcome [216]. Cardiovascular risk factors and comorbid cerebrovascular changes [217] may moderate pharmacological treatment effects or may even have negative effects in the treatment of VaDep [218]. In addition to the appropriate treatment for depression, screening and optimized management of risk factors for cardiovascular and cerebrovascular disease is necessary [219, 220]. Importantly, some data imply that antihypertensive agents, such as beta-blockers, which are widely used by patients with cardiovascular disease, can cause or worsen depression [221]. Nevertheless, controversies still surround these issues, even after decades of widespread use of these drugs [222]. Targeting LLD in individuals with vascular disorders might lower dementia risk by preventing cerebrovascular changes [9]. New methods in management control rely on large datasets (“big data”) of pharmacogenomics, clinical, and pharmacological information and the use of modern mobile applications (apps) for the monitoring of mood and quality of life in individuals is currently in clinical development [223].

## Conclusions

To date, the concept of VaDep is still not widely accepted; there are no formal agreed definitions or diagnostic criteria, the pathomechanisms are not fully understood, the natural history is unknown, and no specific therapy has been confirmed. It is acknowledged that old age depression is a heterogeneous illness with high treatment resistance associated with a number of contributing neurobiological factors, including CVD, neurodegeneration, inflammation, and others, all of which also contribute to its longitudinal prognosis and course [32, 166]. Elderly people are probably vulnerable to depression, and cardiovascular disease, diabetes mellitus, high cholesterol levels, and other such diseases increase the risk for LLD [32, 46, 61, 86, 224]. Drugs used for cardiovascular disease, such as beta blockers, may also potentially cause depression. Thus, if a stroke victim develops depression, this by itself does not prove a causal relationship. VaDep is frequently presumed to be associated with cognitive decline and an increased risk of subsequent dementia [30]. CVD, deep white matter changes, and other (neurodegenerative) lesions have been hypothesized to contribute to increased risk of dementia in the aged, and a host of neuroimaging and clinicopathological studies have examined the interplay between brain pathologies and LLD. This has resulted in new concepts such as the VaDep hypothesis, but despite multiple studies, the relationship between microstructural and related (biochemical) changes in human brain and LLD remains controversial. Recent studies suggested a relationship between brain levels of high-energy phosphate metabolites and executive function in geriatric depression, which is consistent with predictions of the VaDep hypothesis, but further work is necessary to clarify these effects [225]. Unlike VaDep, the hyperfacilitation of the motor cortex found at baseline in vascular MCI-no dementia patients suggested enhanced glutamatergic neurotransmission that might contribute to the preservation of cognitive functioning in these patients [226]. It seems that diagnosing an elderly person as having VaDep just because imaging studies demonstrate WMHs might be debatable, since the latter are quite common in the elderly anyway, particularly in those with cardiovascular disease. Thus, although there is considerable empiric support for the validity of a VaDep subtype of LLD, fundamental questions remain open, including how the illness is defined, how vascular disease and depression influence each other, why VaDep is not a progressive disorder despite the possible related brain lesions tending to accumulate, and whether executive dysfunction or WMHs and global vascular risk are responsible for poor response to anti-depressive treatment [28, 227]. While postmortem findings in some elderly suicided persons revealed lacunes, CSVD, WMHs, and AD-related and other pathologies [228], recent autopsy findings in patients fulfilling the diagnostic criteria of VaDep challenged the role of cerebrovascular pathologies as major morphological substrates of depressive symptoms or poorer executive function and memory in the aged. Similarly, neuropathological data suggested that EOD is not associated with an acceleration of age-related cerebral lesions [31]. Of note, selective serotonin reuptake inhibitor treatment is associated with more neurogenesis and angiogenesis in the human hippocampus [229], whereas in the dentate gyrus, there is less neurogenesis and angiogenesis in MDD patients than in controls. Nevertheless, this trend is reversed by selective serotonin reuptake inhibitor treatment [230], suggesting that one of the mechanisms of action of antidepressants could be through re-establishment of the angiogenesis/neurogenesis niche in this region, which is crucial for memory and emotional regulation. In the case of VaDep drugs, sustaining the vasculature could be essential for cell survival, assuming that vascular changes are the first mediators of cellular changes. There is a need for genetic studies related to cerebral pathologies in LLD in order to better grasp its neuronal basis [231]. Such work may benefit not only from examining genetic markers of neurotransmitter or neuronal activity, but also markers related to vascular disease risk [232].

## Future directions

Genomic signature, neurotrophin and transmitter signaling, neuroinflammation, cerebrovascular lesions, hippocampal neurogenesis, age-related neurodegenerative changes, and other hitherto incompletely elucidated factors may all be involved in the complex pathogenetic cascade that precedes depressive and cognitive symptoms in advanced age. A growing body of evidence from neuroimaging, neurophysiology, and peripheral biomarker studies suggests that depression in old age may be associated with abnormalities in vascular-related and other pathobiological processes [163], but the theory of a distinct subtype of depression named VaDep remains to be fully established.

There are four possible interrelations between cerebrovascular disease and LLD:

1. Depression is the consequence of vascular disease.
2. Depression appears independently from vascular disease, but vascular brain disease may stimulate the development and course of depression.
3. Cerebrovascular pathology and depression may appear without obvious connection as two manifestations of the same genetic predisposition and pathobiological mechanisms.
4. Depression may cause cardiovascular and/or cerebrovascular disease and there may be a bidirectional relationship between depression and vascular disease, but further studies are needed to clarify the mechanisms involved [46].

Thus, to establish a diagnosis of VaDep, it should be based on adequate criteria, such as:

1. Evidence of vascular pathology in elderly subjects with or without cognitive impairment.
2. Absence of previous depressive episodes preceding obvious cerebrovascular disease.
3. Presence of cerebrovascular risk factors.
4. Co-incidence of depression with cerebrovascular risk factors.
5. Clinical symptoms characteristic of VaDep such as executive dysfunction, decrease in processing speed, and lethargy.
6. Neuroimaging data confirming CVD.

However, the temporal relationship between brain pathology and the development of depressive and related symptoms as well as the etiology of VaDep cannot be established on the basis of postmortem observations alone. Therefore, long-term clinicopathological studies, including premortem and postmortem structural MRI, neuropathology, and in vivo functional MRI studies, are warranted in order to further elucidate the relations between structural brain lesions, related pathobiological lesions, and depression in advanced age. Thus far, functional MRI studies have rarely been performed in VaDep, and the few available PET data should be confirmed. In addition to functional MRI, novel techniques, such as more sophisticated PET and combined biomarker studies, may provide better insight into the pathobiological processes involved in mood and cognitive changes in advanced age in order to definitely establish the existence of VaDep and to promote new interventions for its prevention and treatment. These studies might encourage the inclusion of VaDep in future versions of the DSM, setting standards and consensus-approved clinical criteria for the diagnosis of this disorder.

## Abbreviations

AD: Alzheimer’s disease
CSVD: cerebral small vessel disease
CVD: cerebrovascular disease
DSM: Diagnostic and Statistical Manual
EOD: early-onset depression
LLD: late-life depression
LOD: late-onset depression
MCI: mild cognitive impairment
MDD: major depressive disorder
MRI: magnetic resonance imaging
PET: positron emission tomography
SRI: selective serotonin reuptake inhibitor
VaDep: vascular depression
WMH: white matter hyperintensity
